# The Digital Twin in Medicine: A Key to the Future of Healthcare?

**DOI:** 10.3389/fmed.2022.907066

**Published:** 2022-07-14

**Authors:** Tianze Sun, Xiwang He, Xueguan Song, Liming Shu, Zhonghai Li

**Affiliations:** ^1^Department of Orthopedics, First Affiliated Hospital of Dalian Medical University, Dalian, China; ^2^Key Laboratory of Molecular Mechanism for Repair and Remodeling of Orthopedic Diseases, Dalian, China; ^3^School of Mechanical Engineering, Dalian University of Technology, Dalian, China; ^4^Research Into Artifacts, Center for Engineering, School of Engineering, The University of Tokyo, Bunkyo, Japan; ^5^Department of Mechanical Engineering, The University of Tokyo, Bunkyo, Japan

**Keywords:** digital twin (DT), artificial intelligence (AI), precision medicine, healthcare, big data

## Abstract

There is a growing need for precise diagnosis and personalized treatment of disease in recent years. Providing treatment tailored to each patient and maximizing efficacy and efficiency are broad goals of the healthcare system. As an engineering concept that connects the physical entity and digital space, the digital twin (DT) entered our lives at the beginning of Industry 4.0. It is evaluated as a revolution in many industrial fields and has shown the potential to be widely used in the field of medicine. This technology can offer innovative solutions for precise diagnosis and personalized treatment processes. Although there are difficulties in data collection, data fusion, and accurate simulation at this stage, we speculated that the DT may have an increasing use in the future and will become a new platform for personal health management and healthcare services. We introduced the DT technology and discussed the advantages and limitations of its applications in the medical field. This article aims to provide a perspective that combining Big Data, the Internet of Things (IoT), and artificial intelligence (AI) technology; the DT will help establish high-resolution models of patients to achieve precise diagnosis and personalized treatment.

## Introduction

The importance of medical personalization is always understood by Hippocrates’ “There is no disease, there is a patient” (1). The pathogenesis of diseases is increasingly complex and shows obvious differences between different patients. The reasons are the complexity of the genome-wide changes in many diseases and the diagnosis is often delayed because of the late occurrence of symptoms in disease processes that evolve over long periods. Nowadays, most hospital staff can only rely on the traditional knowledge in the field and their basic analysis to plan actions. Customized diagnosis and treatment for patients are the vision of personalized medicine and smart healthcare. The National Institutes of Health (NIH) defined precision medicine as “an emerging approach for disease treatment and prevention that takes into account individual variability in genes, environment, and lifestyle for each person” and physicians are trying to integrate patient-centric data analysis into clinical decision-making (2). However, the initial conception of precision medicine has been criticized for being too centered on genomics and lacking in addressing challenges of clinical management (3). At the same time, there is also a lack of real-time monitoring and crisis warning for patients. The concept is, thus, gradually shifting from the original gene-centric perspective to the wide spectrum of patients’ lifestyles and biological data (4). As simulation plays an increasingly important role in the medical field, so does the feasibility of delivering precision medicine.

The advent of Industry 4.0 has drawn attention to intelligent manufacturing for many years (5). Governments have made efforts to research digitalization and carried out various studies on digital systems, which can remotely manage the business (6). As a virtual replica that models the state of a physical entity or system, the digital twin (DT) acts as a bridge between the physical world and the virtual world, collecting real-time data through sensors, and reflecting them into digital devices. It is increasingly used by companies to improve efficiency both in the production and management (7).

The concept of the DT was first introduced by Professor Michael Grieves in 2002 to describe product life cycle management (8). In its early stage, the National Aeronautics and Space Administration defined it as an integrated multiscale, multiphysics simulation of an as-built vehicle that mirrors the life of the corresponding flying twin in the field of aerospace to predict the life cycle of aircraft (9). Then, wider applications emerged in engineering and manufacturing fields, including simulation, validation, accreditation, etc. (10). In 2018, Cimino et al. (11) described the DT as “a digital twin is a virtual instance of a physical system (twin) that is continually updated with the latter’s performance, maintenance, and health status data throughout the physical system’s life cycle.” In the industrial field, it can build virtual copies of products or workshops through Big Data, which can real-time interact with the deep information and physical space we cannot see normally. Qi et al. (12) put forward the concept of the DT workshop driven by the fused twin data, which can realize iterative operation and optimal management, planning, and control of the workshop. In addition, with the help of artificial intelligence (AI), it can detect possible maintenance needs before machine failures occur through deep learning (13).

The emergency of the DT would make precision medicine possible in the future. Envisaged as a potential solution, the DT technology has already achieved several applications in the medical fields, such as predicting diseases by body examination and reducing errors in medical devices, so as to improve diagnostic accuracy (14). Similar to the engineering products, with the continuous development of the technology of Big Data, the IoT, AI, and sensors, the DT of the human body will have further development and will bring precision medicine into reality (15). This technology offers innovative and definitive solutions for correct diagnosis and following the treatment processes suitable for patients. We propose that the holistic DT of the human body will be created to make a precise diagnosis and real-time monitoring of complex diseases, select appropriate treatment plans, and predict treatment effects, so as to achieve personalized treatment and smart medical care. The application status and further prospect of the DT in the medical field is analyzed in this study.

## Composition and Features of the Digital Twin Technology

### Composition of the Digital Twin Technology

**FIGURE 1:**
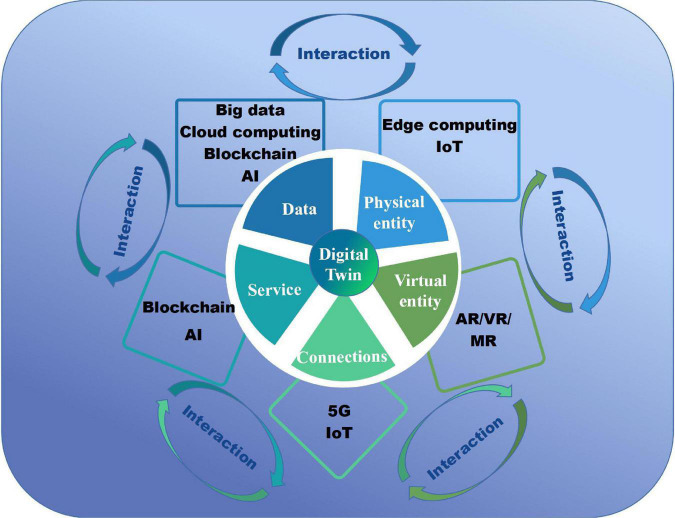
Structural model and technical composition of digital twin.

The DT is constructed in five dimensions, namely, the physical entity, virtual model, connections, the DT data, and service (5). These dimensions are interacted and established the structural model of the DT (Figure 1). The physical entity could be a product, a functional system, or even a city, which performs the defined tasks and collects the data by sensor devices. The IoT provides technical support for the overall perception of the physical entity through data collection methods such as two-dimensional codes, data acquisition cards, and sensors. They are necessary for real-time data collecting and then feedback on the processed data to optimize and regulate the design and operation through communication technology (16). The virtual model is a digital model of the physical entity, which contains variables and abilities of the physical entity and shows its physical properties, geometries, and behaviors in the virtual world. Virtual reality/augmented reality/mixed reality (VR/AR/MR) technology provides support for visualization and fusion of virtual entity and physical entity. The connections realize the interconnection and intercommunication between the various parts of the DT models. 5G communication technology has the characteristics of high speed and low delay, which can meet the needs of digital twin mass data transmission (17). Since the interaction is between the human and the virtual world, human–computer interaction technology, as well as human–robot interaction and collaboration should be considered (18). The DT data includes many categories of information and the fusion between them, enriching the models greatly. Big Data can extract more valuable information from the massive data generated at high speed to explain and predict the results and processes of the DT (19). At the same time, the blockchain can prevent twin data from being tampered with and provide a reliable guarantee for the security of the DT (20). The DT service model includes service for physical entity and virtual model. It improves the fidelity of the virtual model by calibrating parameters and sustaining its performance. AI can automatically perform data analysis, data fusion, and deep learning of twin data through intelligent matching of the best algorithms, thereby greatly improving the value of data and the responsiveness and accuracy of various services.

### Simulation Characteristics of the Digital Twins

High fidelity is the characteristic of simulation and the backbone of the DT, as the accuracy of the simulation and the real-time collection of data have a huge impact on the quality of the DT (21). The data are validated as a benchmark of simulation and calibrated in the continuous field to make the results more accurate. Sensor data and numerical simulation result also ensure the accuracy of the DT. In addition, high standardization, lightweight, robustness, and modularization are necessary conditions for a good virtual model (7). The standardization of communication protocol and encodings can improve the sharing and integration of information and modularity increases the flexibility by recombining or separating individual models (22). These technical features of the DT provide a path to map patient data in the medical process into a predictive framework that combines inductive and deductive reasoning to ultimately achieve precision medicine. Early components of the DTs are already making a clinical impact and the workflow is divided into the stages of data acquisition, diagnosis, and therapy planning. Computational models and statistical models can provide value in those three stages by performing automated image analysis or other operations (23).

### Technical Characteristics of the Digital Twin in the Medical Field

To provide precise diagnosis, virtual fractional flow reserve can replace invasive catheters to monitor arterial blood pressure and body surface recordings that can be mapped to the surface of the heart (24). For example, one approach is operated by using a reduced-order model and machine learning to establish a database that contains computational real blood pressure waveforms. Second, a neural network is built and trained, using easily accessible waveforms as input data to predict unknown blood pressure waveforms. Finally, an additional neural network was trained to analyze the waveform predicted by the inverse model to detect the severity of the disease. The infrastructure of another method consists of offline and online processes. In the offline stage, use the training data and simulation data generated by high-fidelity simulation to construct an efficient AI model and virtual human organs. The online stage is mainly composed of four parts: motion capture system, IK technology, AI model, and visualization. Use motion capture system and IK technology to obtain real-time position and pose of the human body.

## Current Applications of the Digital Twin in Medicine

To realize precision medicine, the core is personalization and patient-centric modeling. With the rapid expansion and developments in many activation technologies, the application of the DT in the medical field shows enormous potential. Due to the fact that the prices of the IoT devices become lower and simpler to implement, the actual network connectivity is magnified (25). The DT healthcare was proposed as a novel medical simulation method to provide robust, precise, and effective medical services using technology combined with multidisciplinary, multiphysics, and multiscale models. We can use AI and other cutting-edge technologies to accurately locate the cause of the patients’ disease, clarify the treatment target, and realize personalized and precise treatment (26). Several preliminary studies have laid a foundation for the further application of the DT in medicine.

### Orthopedics

**FIGURE 2:**
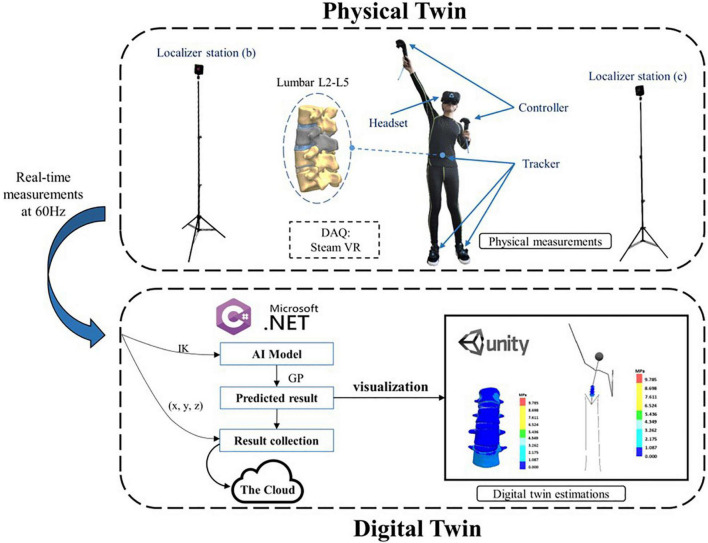
Toward a shape–performance integrated digital twin for lumbar spine analysis (27).

In order to make up for the shortcomings of traditional biomechanical analysis methods in dynamic observation, we carried out the first attempt of the digital twin in orthopedics (Figure 2). Our team built a shape-performance integrated DT body to predict the biomechanical properties of the real lumbar spine under different human postures with the help of customized information collection of the lumbar spine bones of a specific experimenter (27). Based on human motion capture technology, the real-time motion posture and spatial position of the human body were obtained. The lumbar posture of the corresponding human body was calculated according to the wearable virtue reality device and a small amount of sensor data. Using the information of the inverse kinematics system and combined with the finite element method, the DT body of the lumbar spine was established, so as to realize various motion postures of the human body. The AI model performs real-time calculations based on the obtained posture information and the results are fused with the virtual lumbar spine by visualization. In addition, the biomechanical properties of the lumbar spine were evaluated and predicted in real-time and achieved the purpose of real-time monitoring and prediction. Finally, a three-dimensional (3D) virtual reality system was developed with the help of Unity3D software to record the real-time biomechanical performance of the lumbar spine, which could provide a new and effective method of real-time planning in the field of spine treatments. Based on the method for constructing the DT, we have realized the real-time prediction of the intradiskal pressure and the facet contact force, as well as dynamic interaction and connection between physical space and digital space.

To optimize surgical trauma procedures and improve decision-making in postoperative management, Aubert created the DT of a patient’s fracture and modeled four stabilization methods (28). Repeated fracture risks were evaluated regarding the volume of bone with stress above the local yield strength and regarding the interfragmentary strains.

### Cardiovascular Disease

The application of the DT in the cardiovascular system includes the establishment of digital heart models and precise treatment of cardiovascular diseases. Philips developed the personalized DT model based on the unique CT images of the heart, which were obtained before the surgical procedure (29). The tool can provide real-time 3D positioning services to help surgeons locate and select equipment during surgery. Chakshu et al. (30) use a recurrent neural network and propose a methodology for inverse analysis to enable the cardiovascular DT, which can perform inverse analysis with high accuracy. Using easy-accessible arteries blood pressure waveforms such as radial or carotid arteries as input to calculate aortic blood pressure waveforms inversely with the help of deep learning methods. The inverse analysis method makes it possible to develop an active DT that can continuously monitor and prevent the development or further deterioration of medical conditions. With the help of non-invasive or minimally invasive measurement tools, this approach for biomedical applications has the potential to reduce reliance on complex and invasive diagnostic tools.

Jung et al. (31) established a personalized 3D electromechanics model by calibration to clinical cavity pressure data from patients treated for aortic coarctation and generated a high-fidelity model at the cellular scale. It is demonstrated that the multifidelity approach facilitates the personalization of a biophysically detailed active stress model.

### Pharmacy

Dassault Systèmes and the US Food and Drug Administration signed off in 2014 for a project named the SIMULIA Living Heart, which is the first study to look at the organ–drug interactions digitally (32). This is a DT model simulating human hearts and has been validated by researchers or educators in the medical field. With this technology, doctors and pharmaceutical engineers could see the complex structure or the mobility of heart tissue, which will lead to personalized treatment in the future.

Takeda Pharmaceuticals has switched to the DT technology in production to deliver transformative therapies globally. By creating the DT models, it can shorten pharmaceutical processes and make realistic input–output predictions for biochemical reactions (33). Atos and Siemens worked with the pharmaceutical industry to improve the manufacturing process through the physical DT models, which were created to overcome the difficulties in efficiency and production (34). It is currently tested to be successful and is supported by the IoT, AI, and many other advanced technologies.

### Others

Liu et al. (35) proposed a new concept of Digital Twin Healthcare (DTH) in 2019, which is acted as a novel medical simulation method to provide robust, precise, and effective medical services using technology combined with multidisciplinary, multiphysics, and multiscale models. They proposed an effective, highly confidential, and query-like healthcare monitoring plan based on the DT platform. The system consists of three parts: physical object, virtual object, and healthcare data. Each patient is connected to the DT, which can monitor his status and provide strong support in cloud healthcare services for the elderly.

The application of the DT in medicine is mainly focused on chronic disease management. For example, in neurocritical care, current digital technologies focus on interpreting electroencephalogram (EEG), monitoring intracranial pressure, and simulating prognosis (36). It can interpret EEG by helping annotation tracking, detecting seizures, and identifying brain activation in unresponsive patients. In an artificial pancreas model for patients with type 1 diabetes, mathematical models of human glucose metabolism and data algorithms that simulate insulin delivery are customized into the patient-specific DT model, which can continuously calculate insulin requirements and regulate blood insulin concentrations (37, 38). Li et al. presented a scalable framework for modeling and prioritizing disease genes and drug targets among dynamic changes in the DTs for seasonal allergic rhinitis (39).

## Discussion: Present and Future of the Digital Twin in Medicine

### Advantages of the Digital Twin in Medicine

#### Personalized Medicine

The DT is believed to have a positive impact on individual happiness and personalized medicine, enabling patients to exercise a greater degree of autonomy and achieve equitable treatment for patients irrespective of race or gender. The DT makes it possible to develop an active virtual twin that can continuously monitor and prevent the development or further deterioration of medical conditions. In the future, everyone will have his own DT. The holistic integration of the DT is achieved through two complementary pathways: one is driven by personalized mechanistic models provided by key pieces of patient data, in order to refine key decision points in disease management; the other is driven by statistical models powered by electronic records of large populations to optimize the patient’s lifetime journey through the healthcare system.

#### Optimization and Risk Prediction

The development of the DT in the field of medicine is expected to achieve a quantitative understanding and prediction of health and disease, revolutionizing the development of medicine. It can be used in the management and design of the hospital, as well as in the healthcare of patients. Using the DT, various conditions can be predicted and assessed in a virtual environment before scheduling and implementing actual changes, such as bed scheduling and treatment solutions, which will reduce the risks and save costs. It can also transmit information on treatment methods and drugs to the model for verification to optimize the treatment plan and finally realize the early diagnosis or prevention of diseases. Without the DT, hospital staff can only rely on the traditional knowledge of the field and its basic analysis to plan its operations.

#### Monitoring and Precise Treatment

The DT technology is an effective solution for real-time monitoring, precise diagnosis, and treatment. The platform can obtain the real-time health data of patients through wearable devices or mobile phones and analyze possible abnormal conditions in time to realize precise treatment. Using computer algorithm-based methods and principles in bioinformatics, it is possible to select more effective treatment options for the disease according to the DT model of the individual patient, thereby improving the survival rate and quality of the patient’s life.

### Limitations and Challenges

#### Data Collection and Fusion

The acquisition of data is one of the main challenges in both the clinical translation and development of the DT, including geometric data, performance data, sensor data, etc. Nowadays, electronic health records and information are dispersive and difficult to make integrated operations. Unstructured information requires manual work and lacks automation of processing technologies. Due to the inability to obtain real physical monitoring stress data during human movement and difficulties in the fusion of different types of data, there are limitations in building a mature DT model of the human body.

#### Simulation Accuracy

The effectiveness of the technology largely depends to a large extent on the accuracy of simulation, which is another limitation of the application of the DT in the medical field. All the models are simplified representations of physical objects and can be created through a limited scope. To some extent, the results depend on assumptions made. More guidelines, which allow regulators to judge evidence, should be released as a useful tool for building computational models. Other challenges, such as standardization and IT infrastructure, also have to be addressed in the future.

#### Socioethical Risks

There are also socioethical risks in the DT healthcare. Privacy, which seems to be the most important one, is the main reason why the DT may be disadvantageous (40, 41). Furthermore, the high cost of the DT healthcare can lead to inequality and injustice, thereby widening the existing socioeconomic gap. It is assumed that emerging technology is more adjustable in the early stage for the fundamental research and clinical trials have not fully begun and the effects on society are easily manageable (41). Educating citizens or patients on the use and possibilities of the DT technology is a key to its adoption and acceptance. To be sure, healthy people are more worried about their data privacy issues than patients with terminal cancer.

### Prospect

**FIGURE 3:**
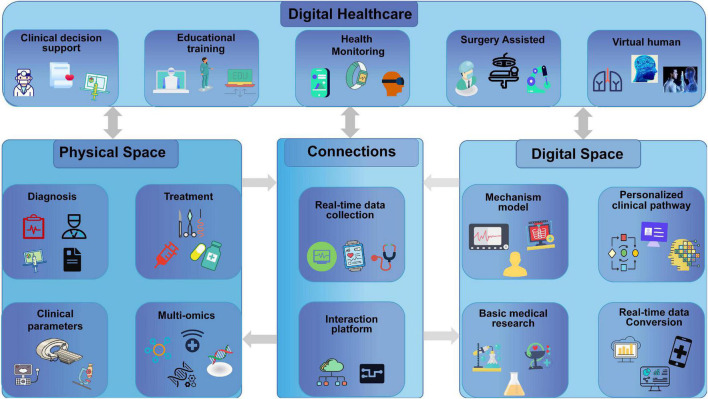
Digital twin in the field of medicine and digital healthcare for personalized treatment.

Combining the DT of medical equipment and medical auxiliary equipment, it will become a new platform and a new experimental method for personal health management and healthcare services. The virtual patient will be perfectly developed by multisource data from medical scanning or wearable instruments, such as CT, MRI, and ultrasound. Biochemical parameters and different types of modules, such as messenger RNAs (mRNAs) and proteins, also help build the DT patient (Figure 3). Furthermore, based on the DT technology and Big Data processing, simulation is performed on the basis of high-resolution models of patients to find accurate treatment targets, suitable drugs or treatment methods for patients to achieve precision medicine, personalized medical treatment, and smart healthcare. Also, the government and international legislatures should conduct strict supervision and establish unified, effective, and feasible standards. The education of citizens, physicians, and researchers on the uses and possibilities of the DT technology is very important for its adoption and acceptance.

Due to the limitations of the existing technologies, there are certain difficulties in digital data acquisition and accurate simulation. The obtaining of real physical monitoring stress data and the fidelity of the finite element model also influenced the accuracy of the DT model. With the advancement of automation technology and the application of Big Data, the DT will establish a standardized application model and a series of problems in healthcare will be better solved through this technology.

## Conclusion

As a virtual replica that models the state of a physical entity, the DT has made a wide application in industry. In the medical field, the DT can help clinicians predict various situations in a virtual environment before implementing actual changes, which will reduce the risks and save costs. Our team first applied this technology to the musculoskeletal system, realizing the dynamic interaction between physical space and digital space. Despite its incomparable advantages in real-time biomechanical analysis, more accurate models, including ligament constraints and soft-tissue material properties, still need to be constructed. Due to the limitations in data collection, data fusion, and accurate simulation, the application of the DT in medicine is not very extensive. With the development of Big Data, the IoT, and AI technology, we propose that the holistic DT of the human body will be created to make real-time monitoring and crisis warning of complex diseases, especially for the elders. All in all, the DT technology could help clinicians make a precise diagnosis, appropriate treatment plans, and predict treatment effects, so as to achieve personalized treatment in the future.

## Data Availability Statement

The original contributions presented in this study are included in the article/supplementary material, further inquiries can be directed to the corresponding author.

## Author Contributions

ZL contributed to the conception and design of the study. TS wrote the first draft of the manuscript. XH, XS, and LS supervised the manuscript. All authors have contributed to the manuscript revision and approved the submitted version of the manuscript.

## Conflict of Interest

The authors declare that the research was conducted in the absence of any commercial or financial relationships that could be construed as a potential conflict of interest.

## Publisher’s Note

All claims expressed in this article are solely those of the authors and do not necessarily represent those of their affiliated organizations, or those of the publisher, the editors and the reviewers. Any product that may be evaluated in this article, or claim that may be made by its manufacturer, is not guaranteed or endorsed by the publisher.
